# Comparative point prevalence survey of antimicrobial consumption between a hospital in Northern Ireland and a hospital in Jordan

**DOI:** 10.1186/s12913-018-3656-y

**Published:** 2018-11-12

**Authors:** Feras Darwish Elhajji, Ghaith M. Al-Taani, Lana Anani, Sahar Al-Masri, Haneen Abdalaziz, Su’ad H. Qabba’h, Abdel Qader Al Bawab, Michael Scott, David Farren, Fiona Gilmore, Ann Versporten, Herman Goossens, Mamoon A. Aldeyab

**Affiliations:** 1 Department of Clinical Pharmacy & Therapeutics, Faculty of Pharmacy, Applied Science Private University, Amman, Jordan; 2 Faculty of Pharmacy, Yarmouk University, Irbid, Jordan; 3 The Pharmacy Department, The Specialty Hospital, Amman, Jordan; 4 Quality & Medication Management, The Specialty Hospital, Amman, Jordan; 5 Su’ad Clinic, Sport City, Amman, Jordan; 6 Faculty of Pharmacy, Al-Zaytoonah University of Jordan, Amman, Jordan; 7 Northern Health and Social Care Trust, Antrim, Ballymena, Northern Ireland, UK; 8 Laboratory of Medical Microbiology, Vaccine & Infectious Disease Institute, Faculty of Medicine and Health Sciences, University of Antwerp, Antwerp, Belgium; 9 School of Pharmacy and Pharmaceutical Sciences, Ulster University, Coleraine, Northern Ireland, UK

**Keywords:** Antimicrobials, Point prevalence survey, Global point prevalence survey, Jordan, Northern Ireland, Antimicrobial surveillance

## Abstract

**Background:**

To assess antimicrobial prescribing in a Northern Ireland hospital (Antrim Area Hospital (AAH)) and compare them with those of a hospital in Jordan (Specialty Hospital).

**Methods:**

Using the Global-PPS approach, the present study surveyed patients admitted to the hospital in 2015, the prescribed antibiotics, and a set of quality control indicators related to antibiotics.

**Results:**

Ultimately, 444 and 112 inpatients in the AAH and the Specialty Hospital, respectively, were surveyed. For the medical group, 165 inpatients were prescribed 239 antibiotics in the AAH, while 44 patients in the Specialty Hospital were prescribed 65 antibiotics. In relation to the surgical group, 34 inpatients treated for infection were prescribed 66 antibiotics in the AAH, while 41 patients in the Specialty Hospital treated for infection were prescribed 56 antibiotics. For the medical patients, the most frequently prescribed antibiotics in the AAH were a combination of penicillins (18.8%) and penicillins with extended spectrum (18.8%). For the surgical patients, the most frequently prescribed antibiotics in the AAH were imidazole derivatives (24.2%). For the medical and surgical patients in the Specialty Hospital, the most frequently prescribed antibiotics were third-generation cephalosporins (26.2 and 37.5%, respectively). In medical patients, compliance to guidelines was 92.2% in the Specialty Hospital compared to 72.0% in the AAH (*p* < 0.001). In surgical patients, compliance to guidelines was 92.7% in the Specialty Hospital compared to 81.8% in the AAH (*p* = 0.012).

**Conclusions:**

The present study highlighted differences in the utilisation of antimicrobials between two hospitals in two distinct regions and benchmarked antibiotic prescriptions across two hospitals.

## Background

Appropriate surveillance and effective infection control practices have been shown to improve hospital-acquired infection and antibiotic prescription [1, 2]. Antibiotics are curative agents directed toward infectious diseases to minimize morbidity and mortality and are considered one of the most important advances in the treatment of medical diseases. The use of antimicrobials, including antibiotics, is increasing worldwide. At the same time, microbial resistance is considered a growing challenge that can render antibiotics ineffective. It is a major health concern that has detrimental effects on society as highlighted via the worrying and alarming levels of antimicrobial resistance, increased mortality, and increased utilization of healthcare resources [3–5]. Healthcare professionals, including pharmacy staff, have identified this challenge and developed antimicrobial stewardship programs. These programs include sets of activities and practices that are directed for the appropriate use of antimicrobials in terms of the selection, dosing, route, and duration of antimicrobial therapy.

The identification and monitoring of antimicrobial consumption and the control of antimicrobial resistance are continuous requirements within the healthcare system. Point Prevalence Surveys (PPSs) provide insights into antibiotic consumption as a surveillance system [3]. Data from such systems can be important in the identification of areas for quality improvement and subsequent interventions to tackle antimicrobial resistance [6–8]. PPSs are a useful and convenient approach for the surveillance of antibiotic consumption. Despite the fact that the continuous surveillance of antibiotic use is ideal, it can be limited because it is a very time and resource consuming approach [7, 9].

PPSs for the surveillance of antibiotics use were utilized to collect data regarding antibiotic use in a number of hospitals internationally. Previous approaches or methods were inadequate as they did not include surgical prophylaxis patients, did not describe the site of infection, and prescribed doses and omitted to mention details about the route of administration. These methods typically surveyed a small number of hospitals (either a single hospital or a regional network of hospitals) [10]. Antimicrobial use studies provided an estimate of the prevalence of antibiotic use ranging from 17 to 49% [2, 11–17].

Based on the broad experience with the European Surveillance of Antimicrobial Consumption (ESAC) [8, 10, 15, 18], the Global Point Prevalence Survey of Antimicrobial Consumption and Resistance (GLOBAL-PPS) project was developed as a surveillance system [19]. The present study aimed to use the GLOBAL-PPS method to assess and compare antimicrobial prescribing in a Northern Ireland hospital, situated in Western Europe, and a hospital in Jordan, situated in the Middle East in Western Asia. According to the antimicrobial consumption overview, antimicrobial policy makers in the Northern Ireland hospital will be expected to compare their results with a reference hospital in not only a different country, but also a different region and a different healthcare system. At the same time, healthcare policy makers in the hospital in Jordan will be expected to adopt an antimicrobial stewardship program based on a tested comparison with a hospital in a developed country.

## Methods

The hospitals included in the present study were secondary care hospitals; the Antrim Area Hospital in Northern Ireland (N. Ireland) and the Specialty Hospital in Jordan. The Antrim Area Hospital (426 beds) is a teaching hospital within the Northern Health and Social Care Trust (NHSCT), which covers acute/emergency hospitalizations in a specific location in Northern Ireland, while the Specialty Hospital (250 beds) is a private teaching hospital in Amman (the national capital of Jordan) that receives emergency and elective admissions from Jordan and nearby Arab countries. Both hospitals have had participated in Global-PPS project, and have been willing to participate in antibiotics-related research.

Data were collected from all wards in both hospitals on a single day in 2015. The survey included inpatients present in each ward at 8 a.m. on the day of PPS. Data sources were mainly chart reviews. Patterns of antimicrobial prescriptions were collected by referral to patients’ case notes and prescription charts by clinical pharmacists. In the event that the information was unavailable from a chart review, the medical or nursing staff were asked for the information.

The standardised Global-PPS protocol allowed collecting data of the number of patients in each ward, demographics (age and sex), specialty, antibiotics used with dosage and route of administration, and site of infection. The survey further included recording the indication of antibiotic prescribing (i.e. hospital-acquired infections, community-acquired infections and surgery or medical prophylaxis). Hospital-acquired infections were defined when the symptoms started two days or more after admission to hospital, and community-acquired infections were recorded in cases where symptoms occurred less than 48 h after admission to hospital [18, 20]. Compliance with guidelines was assessed by referral to local guidelines at each research site and recorded as compliant, non-compliant, non-assessable if no guidelines were existing, or no information if the indication was unclear. Other quality indicators included recording the indication of the antibiotic in patient notes, the documentation of stop/review date, and whether the treatment was based on biomarker data.

Antimicrobials were classified according to the Anatomic Therapeutic Chemical (ATC) classification developed by the World Health Organization (WHO; https://www.whocc.no/atc_ddd_index/). The number of patients prescribed an antibiotic was divided over the total number of inpatients surveyed at ward level in order to calculate the prevalence of antibiotic prescribing. Data were analyzed using SPSS version 21 and included descriptive statistical analyses. Normality of the variables was assessed using Shapiro-Wilk test. Chi square test, independent samples t test and Mann-Whiney U test were used to compare results between Antrim Area Hospital and the Specialty Hospital, according to the distribution of variables. Statistical significance was set at *p* ≤ 0.05.

## Results

The number of surveyed inpatients was 444 inpatients in the Antrim Area Hospital (N. Ireland) and 112 in the Specialty Hospital (Jordan), respectively. In medical and surgical patients, antibiotics were prescribed for 199 patients in Antrim Area Hospital and 85 patients in the Specialty Hospital. Overall (medical, surgical and intensive care), antibiotics were prescribed for 46.2% in the Antrim Area Hospital and 78.2% in the specialty hospital. The mean age of the surveyed patients in the Specialty Hospital was lower than Antrim Area Hospital, e.g., for medical patients the mean age was 70.1 years in the Antrim Area Hospital and 47.6 years in the Specialty Hospital and it was statistically significant (*p* ≤ 0.001). Table 1 summarises the general characteristics of the patients surveyed in both hospitals.

**Table 1:** General characteristics of the patients among the study hospitals by medical and surgical admissions

	Medical			Surgical		
Characteristics	Antrim Area Hospital (*n* (%) of the patients)	Specialty Hospital (*n* (%) of the patients)	*p* value	Antrim Area Hospital (*n* (%) of the patients)	Specialty Hospital (*n* (%) of the patients)	*p* value
Number of treated patients	165	44		34	41	
Age of patients (mean)	70.1	47.6	< 0.001	65.0	43.5	< 0.001
Gender						
- Male	83 (50.3)	29 (65.9)	0.065	12 (35.3)	28 (68.3)	0.004
- Female	82 (49.7)	15 (34.1)		22 (64.7)	13 (31.7)	
Number of prescribed antibiotics^a^	239	65		66	56	
Diagnosis site						
- Central nervous system	4 (2.4)	3 (6.8)	0.112	0 (0.0)	0 (0.0)	0.036
- Eye	0 (0.0)	0 (0.0)		0 (0.0)	1 (2.4)	
- Otolaryngology	4 (2.4)	1 (2.3)		0 (0.0)	0 (0.0)	
- Respiratory	78 (47.3)	15 (34.1)		2 (5.9)	3 (7.3)	
- Cardiovascular	0 (0.0)	1 (2.3)		0 (0.0)	4 (9.8)	
- Gastrointestinal tract	8 (4.8)	6 (13.6)		20 (58.8)	12 (29.3)	
- Skin, soft tissue, bone, and joint	14 (8.5)	6 (13.6)		3 (8.8)	14 (34.1)	
- Urinary tract	21 (12.7)	6 (13.6)		4 (11.8)	4 (9.8)	
- Genito-urinary and obstetrics	10 (6.1)	1 (2.3)		4 (11.8)	2 (4.9)	
- Undefined site	23 (13.9)	5 (11.4)		1 (2.9)	0 (0.0)	
- Neonatal	3 (1.8)	0 (0.0)		0 (0.0)	1 (2.4)	

^a^Some patients were prescribed more than one antibiotic

Antibiotic prescriptions varied between the two study hospitals in terms of type and number. For the medical inpatients, 165 treated for infection were prescribed 239 antibiotics in the Antrim Area Hospital, while 44 patients in the Specialty Hospital treated for infection were prescribed 65 antibiotics. For the surgical inpatients, 34 treated for infection were prescribed 66 antibiotics in the Antrim Area Hospital, while 41 in the Specialty Hospital treated for infection were prescribed 56 antibiotics. For the medical patients, the most frequently prescribed antibiotics in the Antrim Area Hospital were a combination of penicillins (18.8%), penicillins with extended spectrum (18.8%), macrolides (9.6%), tetracyclines (8.8%) and imidazole derivatives (7.5%). For the medical patients in the Specialty Hospital, the most frequently prescribed antibiotics were third-generation cephalosporins (26.2%), fluoroquinolones (18.5%), carbapenems (15.4%), combinations of penicillins (12.3%), and aminoglycosides (9.2%). For the Surgical patients, the most frequently prescribed antibiotics in the Antrim Area Hospital were imidazole derivatives (24.2%), a combination of penicillins (19.7%), aminoglycosides (19.7%) and penicillins with extended spectrum (13.6%). For the surgical patients in the Specialty Hospital, the most frequently prescribed antibiotics were third-generation cephalosporins (37.5%), carbapenems (21.4%) and glycopeptides (12.5%). Significant statistical differences between hospitals achieved for medical patients in prescribing of penicillins with extended spectrum (*p* < 0.001) and third generation cephalosporins (p < 0.001). For surgical patients, significant statistical difference between hospitals achieved in prescribing of imidazole (*p* = 0.001) and third generation cephalosporins (*p* < 0.001). Table 2 provides full details regarding the antibiotic agents prescribed in both hospitals.

**Table 2:** Antibiotic agents prescribed among the study hospitals (% of prescribed antibiotics)

	Medical			surgical		
Antibiotic prescriptions (ATC4)	Antrim Area Hospital (*n* = 239 antibiotic prescriptions) n (%)	Specialty Hospital (*n* = 65 antibiotic prescriptions) n (%)	*p* value	Antrim Area Hospital (*n* = 66 antibiotic prescriptions) n (%)	Specialty Hospital (*n* = 56 antibiotic prescriptions) n (%)	*p* value
Intestinal anti-infectives (antibiotics) (A07AA)	6 (2.5)	0 (0.0)	0.197	0 (0.0)	0 (0.0)	n/a
Tetracyclines (J01AA)	21 (8.8)	3 (4.6)	0.269	1 (1.5)	2 (3.6)	0.465
Penicillins with extended spectrum (J01CA)	45 (18.8)	0 (0.0)	< 0.001	9 (13.6)	0 (0.0)	0.004
Beta-lactamase sensitive penicillins (J01 CE)	4 (1.7)	0 (0.0)	0.294	0 (0.0)	0 (0.0)	n/a
Beta-lactamase resistant penicillins (J01CF)	10 (4.2)	0 (0.0)	0.094	2 (3.0)	0 (0.0)	0.189
Combinations of penicillins, including b-lactamase inhibitors (J01CR)	45 (18.8)	8 (12.3)	0.219	13 (19.7)	4 (7.1)	0.046
First-generation cephalosporins (J01DB)	1 (0.4)	1 (1.5)	0.322	1 (1.5)	2 (3.6)	0.465
Second-generation cephalosporins (J01 DC)	4 (1.7)	0 (0.0)	0.294	0 (0.0)	2 (3.6)	0.122
Third-generation cephalosporins (J01DD)	5 (2.1)	17 (26.2)	< 0.001	0 (0.0)	21 (37.5)	< 0.001
Monobactams (J01DF),	7 (2.9)	0 (0.0)	0.163	0 (0.0)	0 (0.0)	n/a
Carbapenems (J01DH)	3 (1.3)	10 (15.4)	< 0.001	1 (1.5)	12 (21.4)	< 0.001
Trimethoprim and derivatives (J01EA)	4 (1.7)	0 (0.0)	0.294	1 (1.5)	0 (0.0)	0.355
Combination of trimethoprim/sulfamethoxazole (J01EE)	6 (2.5)	0 (0.0)	0.197	0 (0.0)	0 (0.0)	n/a
Macrolides (J01FA)	23 (9.6)	3 (4.6)	0.201	0 (0.0)	0 (0.0)	n/a
Aminoglycosides (J01GB)	11 (4.6)	6 (9.2)	0.150	13 (19.7)	1 (1.8)	0.002
Fluoroquinolones (J01MA)	3 (1.3)	12 (18.5)	< 0.001	2 (3.0)	3 (5.4)	0.518
Glycopeptide antibacterials (J01XA)	12 (5.0)	2 (3.1)	0.507	4 (6.1)	7 (12.5)	0.216
Steroid antibacterials (J01XC)	1 (0.4)	0 (0.0)	0.601	0 (0.0)	0 (0.0)	n/a
Imidazole derivatives (J01XD)	18 (7.5)	2 (3.1)	0.199	16 (24.2)	2 (3.6)	0.001
Nitrofuran derivatives (J01XE)	2 (0.8)	0 (0.0)	0.459	0 (0.0)	0 (0.0)	n/a
Other antibacterials (J01XX)	0 (0.0)	1 (1.5)	0.055	1 (1.5)	0 (0.0)	0.355
Antimycotics, triazole derivatives (J02 AC)	2 (0.8)	0 (0.0)	0.459	0 (0.0)	0 (0.0)	n/a
Antimycotics, other antimycotics for systematic use (J02AX)	1 (0.4)	0 (0.0)	0.601	0 (0.0)	0 (0.0)	n/a
Antimycobacterials, antibiotics (J04AB)	5 (2.1)	0 (0.0)	n/a	0 (0.0)	0 (0.0)	n/a
Antivirals, neuraminidase inhibitors (J05AH)	0 (0.0)	0 (0.0)	n/a	0 (0.0)	0 (0.0)	n/a
Antiprotozoals, nitroimidazole derivatives (P01AB)	5 (2.1)	1 (0.8)	0.240	2 (3.0)	0 (0.0)	0.189

n/a: not applicable

As described in Table 3, the most common indication for prescribing antibiotics for medical patients in both hospitals was community-acquired infection (71.1% for the Antrim Area Hospital *vs.* 60.0% in the Specialty Hospital), in which no statistical differences were noted between the hospitals. Among the surgical patient the most common indication for prescribing antibiotics in the Antrim Area Hospital was community-acquired infection (56.1%), while for the Specialty Hospital it was surgery prophylaxis (89.2%). Statistical significant differences between Antrim Area Hospital and specialty hospital for the following indication groups, including surgery prophylaxis (medical and surgical; both p < 0.001); medical prophylaxis (medical; p < 0.001) and community acquired infections (surgical; p < 0.001). Among medical, surgical and intensive care patient in the Antrim Area Hospital, the most commonly prescribed antibiotic for community- and hospital-acquired infections was a combination of penicillins (J01CR) but for surgical prophylaxis it was imidazole derivatives (J01XD). Overall, in the Specialty Hospital, the most commonly prescribed antibiotic for community-acquired infection and medical or surgery prophylaxis was third-generation cephalosporins (J01DD).

**Table 3:** Antibiotics prescribed by indication groups among the study hospitals

	medical			surgical		
Indication group	Antrim Area Hospital (*n* (%); 239 antibiotic prescriptions)	Specialty Hospital (*n* (%); 65 antibiotic prescriptions)	*p* value	Antrim Area Hospital (*n* (%);66 antibiotic prescriptions)	Specialty Hospital (*n* (%); 56 antibiotic prescriptions)	*p* value
Community-acquired infection (CAI)	170 (71.1)	39 (60.0)	0.086	37 (56.1)	5 (8.9)	< 0.001
Hospital-acquired infection (HAI)	43 (18.0)	0	n/a	6 (9.0)	0	n/a
Surgery prophylaxis	10 (4.2)	11 (16.9)	< 0.001	21 (31.8)	50 (89.2)	< 0.001
Medical prophylaxis	15 (6.3)	15 (23.1)	< 0.001	2 (3.0)	1 (1.8)	0.653

n/a: not applicable

In medical patients, compliance with guidelines was 92.2% in the Specialty Hospital compared to 72.0% in the Antrim Area Hospital. Among the surgical patients, compliance to guidelines was 92.7% in the Specialty Hospital compared to 81.8% in the Antrim Area Hospital. Reasons for antibiotic prescription were documented in 100 and 94.1% of the notes for the medical patients in the Specialty Hospital and the Antrim Area Hospital, respectively. For the surgical patients, reasons for antibiotic prescription were documented in 98.2 and 83.3% of the notes in the Specialty Hospital and the Antrim Area Hospital respectively. For approximately half of the inpatients surveyed in both hospitals, the stop/review date was documented in the medical notes. Surveyed medical inpatients at the Antrim Area Hospital had their treatment prescribed based on biomarker data in 70.3% of cases, compared to 53.8% at the Specialty Hospital. Among surgical patients, 42.4% of the patients in Antrim Area Hospital had their treatment prescribed based on biomarker data versus 28.6% of the patient in the Specialty Hospital (Table 4). For the medical patients, statistical significant differences between the two hospital were noted in relation to compliance with guidelines (*p* < 0.001), documenting the reason for prescribing (*p* = 0.045), and treatment based on biomarker data (*p* = 0.001). Whereas for surgical patient, statistical significant differences between the two hospitals were noted in relation to compliance with guidelines (*p* = 0.012), documenting the reason for prescribing (*p* = 0.006), and stop/review date documented (*p* = 0.080; Table 4).

**Table 4:** Targets for quality control among study hospitals

		Medical			Surgical		
Group		Antrim Area Hospital (239 antibiotic prescriptions); *n* (%)	Specialty Hospital (65 antibiotic prescriptions); *n* (%)	*p* value	Antrim Area Hospital (66 antibiotic prescriptions); *n* (%)	Specialty Hospital (56 antibiotic prescriptions); *n* (%)	*p* value
Compliance with guidelines (% of prescribed antibiotics)	Compliant	172/239 (72.0%)	59/64 (92.2%)	< 0.001	54/66 (81.8%)	51/55 (92.7%)	0.012
	Not compliant	45/239 (18.8%)	1/64 (1.6%)		10/66 (15.2%)	0	
	Non assessable	22/239 (9.2%)	4/64 (6.3%)		2/66 (3.0%)	4/55 (7.3%)	
	No information	0	1/65 (1.5%)		0	1/56 (1.8%)	
Reason for prescribing antibiotic is documented		225 (94.1%)	65 (100%)	0.046	55 (83.3%)	55 (98.2%)	0.006
Stop/review date is documented		125 (52.3%)	31 (47.7%)	0.510	32 (48.5%)	36 (64.3%)	0.080
Treatment based on biomarker data (e.g., C reactive protein)		168 (70.3%)	35 (53.8%)	0.001	28 (42.4%)	16 (28.6%)	0.112

## Discussion

To help addressing the challenge of antibiotic resistance, surveillance of antibiotic use is a continuous requirement. An antimicrobial stewardship program is considered an initiative that aims to combat antimicrobial resistance [21]. A PPS of antimicrobial consumption has been utilised internationally to collect accurate, inexpensive data regarding antibiotic consumption that are useful for setting priorities to promote prudent antimicrobial use [6–8]. At the same time, PPS has been applied to monitor adherence to antimicrobial prescription protocols and identify targets for quality improvement [22–24]. The present study describes trends in antimicrobial prescribing practices between two hospital settings within Northern Ireland and Jordan, two countries located in different regions.

In the present study the prevalence of antimicrobial agents prescribed was 46.2% for the Antrim Area Hospital in Northern Ireland and 78.2% for the Specialty Hospital in Jordan. A number of studies have addressed PPS from the Northern Ireland hospital setting (four acute care hospitals), finding that the prevalence of prescribing antibiotics had ranged from 31 to 36% in May 2008 and May–June 2009, respectively [25, 26], which is consistent with the prevalence of antimicrobial use within European countries (34.6%) [19, 27] within approximately the same period. It is unclear why we observed a higher prevalence of antibiotic prescribing in the Antrim Area Hospital in 2015. No published data on the prevalence of antimicrobial prescribing for hospitalised patients in Jordan could be found, meaning no reference number exists. Therefore, the current results can be considered as a benchmark for antibiotics stewardship in Jordan, at the same time it can give a first impression about a relatively higher tendency towards prescribing antibiotics. Figures for the prevalence of antimicrobial drugs to hospitalised patients, utilising PPS, varied between different countries. For example, antimicrobial prescription rates observed in a number of hospitals were 59% in Egypt, 42% in India, 22% in Holland, 49% in Italy, and 50% in the USA [28–32].

Some major differences were found in the general and clinical characteristics between the two hospitals examined in this study, such as the higher age of the patient sample in the Antrim Area Hospital and a higher proportion of surgical patients to medical patients in the Specialty Hospital compared to the Antrim Area Hospital. These differences can be attributed to differences between both settings, particularly in relation to the demographics and types of patient population each hospital serves. Such a high proportion of surgical patients in the Specialty Hospital might explain, to some degree, the high rate of antibiotics prescribing (for surgery prophylaxis) compared to the Antrim Area Hospital, as the majority of patients in the latter hospital were medical patients.

The most common group of antimicrobial agents prescribed in the Antrim Area Hospital was penicillins with extended spectrum and combination of penicillins, while the Specialty Hospital prescribed considerably more broad spectrum antibiotics, namely, third-generation cephalosporins, fluoroquinolones and carbapenems. One published Jordanian study addressed substantial concerns regarding multi-drug resistant isolates for carbapenems [33]. The difference in types of the prescribed antimicrobials was obvious and significant, reflecting the nature of the used local guidelines in both hospitals.

First-generation cephalosporins (e.g. cefazolin) are usually recommended as prophylactic antibiotics for many surgical procedures. Considering that around two-thirds of the patients in the Specialty Hospital were in the surgical prophylaxis group, the rate of prescribing of such antibiotics is expected to have been relatively high. Yet, only 3.6% of the studied patients in the Specialty Hospital received first-generation cephalosporins. This could indicate a tendency among surgeons to utilise broader-spectrum antibiotics, which can increase drug resistance levels and drug utilisation costs. In fact, this can be considered an area for improvement that requires efforts in the future. Ideally, broad spectrum antibiotics should be reserved for use in cases of severe infection as the indiscriminate use of these agents can be associated with increased risk of antimicrobial resistance development [34].

The PPS proved useful for the collection of quality control data related to antimicrobial use and identifying targets for quality control. Both hospitals included in this study are deemed in compliance with antibiotic guidelines and the documentation of the reason for prescribing antibiotics. In relation to the compliance with guidelines, the 72.0% compliance with antimicrobial guidelines (medical patients) for the Antrim Area Hospital is consistent with other studies with similar healthcare systems—namely, 59.7% in Australia, 90% in Scotland, and 75.7% in the Netherlands [22, 35, 36]. The reason for prescribing antibiotics for medical patients was recorded in 94.1% of the patients’ notes in the Antrim Area Hospital and 100% in the specialty hospital. Such an effect could be attributed to the availability of antimicrobial stewardship programs in the Antrim Area Hospital and the presence of local guidelines in the Specialty Hospital.

The present study is limited because it did not take into account the effect of the case-mix and comorbidities that the surveyed patients had, which could affect the prescribing pattern of antibiotics. As such, the Specialty Hospital in Jordan serves a diversity of patients from different developing countries where resistance to antibiotics is high due to irrational use. Guideline compliance (which referred to drug choice only) could be a result of increased documentation of the reason for prescribing an antibiotic.

## Conclusion

The present study highlighted differences in the utilisation of antimicrobials between two hospitals in two distinct regions (Antrim Area Hospital in Northern Ireland and Specialty Hospital in Jordan). A higher rate of prescribing antibiotics was noted in the Specialty Hospital compared to the Antrim Area Hospital. More broad spectrum antibiotics (e.g. third-generation cephalosporins) were utilised in the Specialty Hospital than in the Antrim Area Hospital. The present study was able to benchmark antibiotic prescriptions across the two hospitals, which could improve the use of antibiotics and prescribing practices through dedicated antibiotic stewardship programs.

## Abbreviations

AAH: Antrim Area Hospital
ATC: Anatomic Therapeutic Chemical
ESAC: European Surveillance of Antimicrobial Consumption
NHSCT: Northern Health and Social Care Trust
PPS: Point Prevalence Survey
WHO: World Health Organization
